# Is the quality of primary healthcare services influenced by the healthcare centre’s type of ownership?—An observational study of patient perceived quality, prescription rates and follow-up routines in privately and publicly owned primary care centres

**DOI:** 10.1186/s12913-015-1082-y

**Published:** 2015-09-26

**Authors:** Andy Maun, Catrin Wessman, Pär-Daniel Sundvall, Jörgen Thorn, Cecilia Björkelund

**Affiliations:** Department of Medicine, Division of General Practice, University Medical Centre Freiburg, Elsässerstr. 2 m, D-79110 Freiburg, Germany; Institute for Quality Management and Social Medicine, University Medical Centre Freiburg, Engelbergerstr. 21, D-79106 Freiburg, Germany; Department of Public Health and Community Medicine/Primary Health Care, Institute of Medicine, The Sahlgrenska Academy, University of Gothenburg, Box 454, SE-405 30 Göteborg, Sweden; Centre for Applied Biostatistics, The Sahlgrenska Academy, University of Gothenburg, Box 414, SE-405 30 Göteborg, Sweden; Research and Development Unit, Primary Health Care in Southern Älvsborg County, Sven Eriksonsplatsen 4, SE-503 38 Borås, Sweden

**Keywords:** Health services research, Primary healthcare, Privatisation, Public sector, Private sector, Government policy, Reform, Quality improvement, Economic competition, Scandinavia

## Abstract

**Background:**

Primary healthcare in Sweden has undergone comprehensive reforms, including freedom of choice regarding provider, freedom of establishment and increased privatisation aiming to meet demands for quality and availability. In this system privately and publicly owned primary care centres with different business models (for-profit vs non-profit) coexist and compete for patients, which makes it important to study whether or not the type of ownership influences the quality of the primary healthcare services.

**Methods:**

In this retrospective observational study (April 2011 to January 2014) the patient perceived quality, the use of antibiotics and benzodiazepine derivatives, and the follow-up routines of certain chronic diseases were analysed for all primary care centres in Region Västra Götaland. The outcome measures were compared on a group level between privately owned (*n* = 86) and publicly owned (*n* = 114) primary care centres (PCC).

**Results:**

In comparison with the group of publicly owned PCCs, the group of privately owned PCCs were characterized by: a smaller, but continuously growing share of the population served (from 32 to 36 %); smaller PCC population sizes (avg. 5932 vs. 9432 individuals); a higher fraction of PCCs located in urban areas (57 % vs 35 %); a higher fraction of listed citizens in working age (62 % vs. 56 %) and belonging to the second most affluent socioeconomic quintile (26 % vs. 14 %); higher perceived patient quality (82.4 vs. 79.6 points); higher use of antibiotics (6.0 vs. 5.1 prescriptions per 100 individuals in a quarter); lower use of benzodiazepines (DDD per 100 patients/month) for 20–74 year olds (278 vs. 306) and >74 year olds (1744 vs.1791); lower rates for follow-ups of chronic diseases (71.2 % vs 74.6 %). While antibiotic use decreased, the use of benzodiazepines increased for both groups over time.

**Conclusions:**

The findings of this study cannot unambiguously answer the question of whether or not the quality is influenced by the healthcare centre’s type of ownership. It can be questioned whether the reform created conditions that encouraged quality improvements. Tendencies of an (unintended) unequal distribution of the population between the two groups with disparities in age, socio-economy and geography might lead to unpredictable effects. Further studies are necessary for evidence-informed policy-making.

## Background

From the perspective of citizens and patients, it is most important that healthcare reforms lead to an efficient use of resources with access to healthcare that is knowledge-based, efficient, safe, patient-focused, equitable and timely [1]. In order to meet demands for quality and availability in primary healthcare, Sweden has carried out comprehensive reforms in recent years, including freedom of choice regarding provider, freedom of establishment and increased privatisation [2].

The Swedish health care system is a socially responsible system with an explicit public commitment to ensure the health of all citizens and follows the principles of human dignity, need and solidarity, and cost-effectiveness [3]. Health care expenditure in Sweden is mainly tax funded (80 %), user charges for visits to professionals, hospitalization and medicines fund about 17 % and primary care constitutes for around 20 % of the total healthcare budget [3]. Primary care in Sweden is delivered by more than 1100 publicly and privately owned primary care centres (PCC) throughout the country.

The recent reform means that the County Councils only define the assignment and reimbursement schemes and may not decide who is to provide care or where it will be carried out. It also means that providers that fulfil general quality requirements have the freedom of establishment (without any geographical restrictions or limitations in numbers) and that the providers are competing for patients. The aim of these reforms were, according to the government, to focus on the individual and to shift power away from politicians and officials to citizens, thus increasing citizens’ choice and influence as well as increasing the number of providers and their diversity. The government argued that the reforms would create conditions that encourage care providers to improve the quality and efficiency of care, as the compensation comes with the patients who will seek the best provider according to their preferences. All PCCs have to ensure equal access to their listed citizens according to their medical conditions and cannot refuse citizens to register. Individuals seeking care should be able to get in touch with the PCC the same day (availability guarantee) and should be given an appointment with a doctor within 7 days from the time of initial contact, provided that the caregiver has determined that the person needs to visit a doctor (visiting warranty). Citizens can at any time without any restrictions choose to leave a PCC and register at another PCC of their choice and may not be rejected [4]. In the majority PCCs are either publicly owned by the primary care organisations of the county councils or privately owned by healthcare corporations, only a minority of PCCs are privately owned by their operators. The absolute majority of the numerous recently established PCCs are privately owned [4]. Each provider organization decides independently the rate of the salary and working conditions such as the number of patients or patient visits per General Practitioner (GP). Due to a lack of GPs in Sweden there is also a recruitment competition between primary care centres. Regardless of their ownership type all PCCs in a county are compensated using the same model. Each of the provider organisations or corporations has solely financial responsibility for their PCCs. Providers may support units that go in debt within their organsation or, if accessible, through external financial support e.g. investments of their shareholders. In the studied Region Västra Götaland the compensation for PCCs is mainly based on capitation (90 %), with adjustments for age, gender and the morbidity burden of their patient population as determined by the John Hopkins Adjusted Clinical Groups System based on the registered diagnoses for the individual patient [5]. The remaining 10 % of the compensation is based on geographical and socioeconomical factors, structural goals, patient-satisfaction and pay-for-performance schemes (3 %). The compensation has to cover operating costs of the PCC including the salaries, the budget for prescribed medications and diagnostic tests such as laboratory and radiologic examinations. If profits are generated after deduction of costs, publicly owned provider organisations have to reinvest them in their organisation, while privately owned provider organisations can pay out a divident to their shareholders.

As the aims of these reforms have been to strengthen the role of the patient and to improve accessibility and quality, the Swedish Association of Local Authorities and Regions (SALAR) annually conducts a national patient survey and publishes the results for each PCC in the country. Moreover a number of studies have been carried out to monitor the ambiguous effects of the reforms. About 60 % of the population in three larger Swedish counties perceived that they had made a choice of provider after the introduction of the reforms [6]. There were no clear signs of absolute displacement effects, i.e. that certain patient groups increased their utilisation of health services while others reduced it, but the population as a whole had increased its utilisation to a greater extent than people with major care needs [7]. Primary care managers of publicly owned centres found it more difficult to prioritise correctly between patient groups with different needs, demands and levels of empowerment and were concerned about potentially negative effects on less empowered persons [8]. While the general public and the patients attending primary care scored higher satisfaction rates after the reforms–people with higher incomes in particular showed higher satisfaction rates with the development of accessibility–staff experienced a certain degree of deterioration [7, 9].

In this environment with strong economic incentives and an intense competition among PCCs for listed patients and in the recruitment of GPs, medical decisions might be compromised for economic reasons. Additionally the structural difference between coexisting non-profit and for-profit organisations has to be considered, which has been object of earlier controversies criticizing higher costs and lower quality of for-profit organisations [10–12]. As earlier studies showed effects of financial incentives on medical practice and suggest to rigorously evaluate the impact of changes in the payment system, these conditions make it important to study whether or not the quality of the primary healthcare services available is influenced by the PCCs’ type of ownership [13, 14].

This study focuses on outcomes that possibly can be influenced by the given incentives. The assumption is that PCCs are likely to strive after high patient satisfaction to maintain or increase the numbers of patients listed. However, research has shown that higher patient satisfaction can be associated with higher overall health care and prescription drug expenditures and paradoxically can even be associated with increased mortality [15].

Despite the fact that an increased use of antibiotics heightens the probability of antibiotic resistance in the population, a prior study showed that patients’ expectations had a significant influence on the prescription of antibiotics. Patients who were not prescribed an antibiotic that they wanted were more likely to be dissatisfied and to re-consult twice [16, 17]. One concern is that physicians might be influenced in their medical decisions in order to avoid either more frequent re-consultation by dissatisfied patients (without being reimbursed for that effort) or that physicians fear that patients may choose to register themselves at another primary healthcare centre. Similar effects could be apprehended for the prescription rates of benzodiazepine derivatives: despite the fact that these are only recommended for short periods of treatment and regular use is associated with negative health effects such as addiction [18], prescription rates in the county studied have been the highest in the country for decades and have exceeded US levels threefold [19]. The assumption is physicians might be influenced in their medical decisions when conflicts of interest between the physician and the patient arise that can be time-consuming or have an impact on the patient’s satisfaction and choice of the primary care provider. As the compensation is partly based on the morbidity burden documented by the registered diagnoses for the individual patient, it is also of interest to study and compare the rates of follow-ups actually carried out for chronic conditions where reliable information is available.

The aim of this study is to compare privately and publicly owned PCCs in Region Västra Götaland in Sweden on a group level concerning the quality of care provided. The study focuses on patient perceived quality, rates of purchased prescriptions of antibiotics and benzodiazepine derivatives as well as the percentage of follow-up routines carried out for patients with the chronic diseases diabetes mellitus, chronic ischemic heart disease and hypertension.

## Methods

### Study design

This study is a descriptive analysis of all accessible PCCs in Region Västra Götaland. Hence it is a retrospective observational cohort study at the level of PCC. Quality standards for observational studies were assured through the application of the STROBE 22-item checklist (items addressed below) [20].

### Eligibility criteria

All contracted PCCs located in Region Västra Götaland during the period between April 2011 and January 2014 were included. In January 2014 there were 201 PCCs (approved by the regional healthcare authorities) that served 99.9 % of the population of 1.6 million citizens. The small number of single-handed practices serving a very small proportion of the population on a regular basis (<1 %) and Out-of-hours services organised in clusters of PCCs were excluded from the analysis because they had particular assignments and very little data for these activities was available.

### Data sources and data collection

All datasets were publicly available from the regional healthcare authorities [21] and were already aggregated on PCC level and thus do not contain data on the level of the individual citizen. The datasets included information on productivity and a number of quality outcome measurements which the authority collected from the PCCs’ electronic administrative systems, the administrative agency Statistics Sweden [22], the national patient survey [23], the National Prescribed Drug Register [24], the Swedish eHealth Agency [25], the National Diabetes Register (NDR) [26] and the regional Quality Registry for chronic diseases (QregPV) [27]. As all PCCs have only been obliged to approve data collection by the regional healthcare authorities since April 2011, no data prior to that was available. All datasets were either on a monthly or annual basis.

### Variables

The following datasets of independent variables of the PCCs were included in the study:

1. The ownership type of the PCC: either privately or publicly owned (completeness rate 100 %).
2. The geographical location of the PCC: as earlier research indicated structural differences in primary care in terms of density and new establishment rates of PCC and patient perceived quality [4, 28] between densely and more sparsely populated areas the research team divided all PCCs into two groups; either belonging to the metropolitan area of Gothenburg (within 20 km range) or not (completeness rate 100 %).
3. The number of citizens listed at the PCC on an average yearly basis (completeness rate 100 %).
4. The proportion of female and male citizens listed at the PCC on a yearly basis (completeness rate 100 %).
5. The proportional size of the three different age groups (aged below 20, aged 20–64 and aged 65 and above) of citizens listed at the PCC on a yearly basis. As data was only available in age groups at 5-year intervals the research team chose to combine the data into three groups that have qualitatively different primary care needs (completeness rate 96.6 %).
6. The socioeconomic index of the listed population on an average yearly basis: as prior research has shown that PCC populations differ greatly in socioeconomic status, which has an impact on the prevalence of multi-morbidity on a group level, the Care Need Index (CNI) for each PCC was included [29, 30]. The administrative agency Statistics Sweden regularly calculates the CNI for each PCC by the regionally-adjusted factors age, family status, educational status, employment status and migration status (completeness rate 100 %).

The following datasets of dependent variables of the PCCs were included in the study:

1. Patient Perceived Quality: The annual results of the national patient survey (NPS) 2011–2013 expressed by the weighted Patient Perceived Quality (PPQ) values between 0 (minimum) and 100 (maximum) [23]. SALAR randomly selected annually 39,000 patients in the Västra Götaland Region who visited a PCC in September and sent them a letter with questions concerning perceived service, participation, information, accessibility, confidence, usefulness, recommendation, and overall impression. The research team selected the four variables from the NPS considered most relevant for this study: the mean PPQ value; the value that measures to what extent the patient would recommend the PCC to other patients; the value that measures the perceived accessibility to the PCC; and the value that measures the perceived interpersonal continuity. However, even if the completeness rate of datasets for all PCCs was high (97.4 %) it has to be considered that the response rates of the annual NPS were between 51.3 and 53.4 % and that results are negatively confounded in socio-economic disadvantaged and urban districts. Therefore the results of the NPS have to be interpreted with caution.
2. Rate of purchased antibiotics prescribed at a PCC: The Public Health Agency of Sweden and the Swedish Strategic Programme Against Antibiotic Resistance (Strama) continuously follow up the total antibiotic use in the population on a monthly, quarterly, and yearly basis [31]. This study does not count the actual number of prescriptions made at a PCC but uses a proxy measure to describe the antibiotic use at a PCC: through data from the Swedish eHealth Agency the number of purchased antibiotics per 100 individuals listed at a PCC is calculated for each quarter of a year. Due to permanent changes in the number of listed individuals this quote is calculated for each month of a quarter. Drugs with the Anatomical Therapeutic Chemical classification (ATC) code J01 are included in this follow-up, except for the antiseptic substance methenamine hippurate J01XX05 which is not an antibiotic and has no influence on antibiotic resistance. Throughout this paper, methenamine hippurate is consequently excluded whenever antibiotics are referred to. This proxy measure for antibiotic use is neither adjusted for age nor gender, nor is it necessarily connected to the patients listed at the PCC. (Completeness rate 95.6 %).
3. Rate of purchased benzodiazepine derivatives prescribed at a PCC: the rate for the monthly use of benzodiazepine derivatives is expressed by the quotient of defined daily doses (DDD) [32] per 100 listed individuals visiting the PCC in the same month. The DDD is based on the assumed average dose per day for the drug given to adults for its main indication. The calculation included the DDD of prescribed benzodiazepine derivatives with the ATC codes N05BA, N05CD, N05CF that were purchased at pharmacies. As the rates were already divided into subgroups for younger (20–74 years old) and older (>74 years old) patients by the regional healthcare authority (because rates differ considerably for these two age groups) these age strata differ from the rest of the analysis (completeness rate 95.4 %) [18].

As only reliable data was available for patients with diabetes mellitus (DM), ischemic heart disease (IHD) and hypertension (HPT) concerning follow-up routines that had been carried out, only these chronic conditions were included in the study.

4. Annual rate of follow-up routines that were carried out for patients of all age groups with DM: the rate was calculated through the quotients of measurements documented at least once annually for glycated haemoglobin (HbA1c), blood pressure, body mass index (BMI), smoking habits, urine micro-albumin and low-density lipoprotein (LDL) and the number of patients with the diagnosis DM (completeness rate 99.6 %).
5. Annual rate of follow-up routines that were carried out for patients of all age groups with IHD: the rate was calculated through the quotients of smoking habits and measurements of blood pressure and LDL documented at least once annually and the number of patients with the diagnosis IHD. Data was only available for 2012 (completeness rate 89.5 %).
6. Annual rate of follow-up routines that were carried out for diagnosed HPT for all patients aged 18–79 with solely HPT (absence of DM or IHD): the rate was calculated through the quotients of measurements documented at least once annually for blood pressure and smoking habits and the number of patients with the diagnosis HPT. Data was only available for 2012 (completeness rate 97.1 %).

Due to technical or other reasons unknown to the research group at the regional healthcare authorities during the collection of data from the above-mentioned sources, minor percentages of datasets were missing. For integrity reasons the NPS did not publish data for PPQ when the total number of individuals in a dataset was very low (*n* < 30). The research group did not actively exclude any of the data received.

As all data reflects only calculated measures of the actual month or year for each PCC, data from PCCs that closed down or were newly-opened was included during the time of their existence.

All PCCs were divided into the two groups of privately and publicly owned PCCs. In some contexts further sub-grouping into socioeconomic quintiles or geographical location was applied.

### Data analysis of the total population

Descriptive statistic calculations of the independent variables (sample sizes and demographic characteristics expressed through mean values and standard deviations (SD)) were conducted for the groups of privately and publicly owned PCCs respectively including the changes over time. All data analysed was already aggregated at the PCC level-where the new economic incentives applied to all units in exactly the same way-using the same methods and time intervals for data collection with very low fractions of missing data. As the analysis carried out was a study of a total population with very high rates of data completeness, no power calculation and/or tests for statistical significance were performed. Instead the mean values and standard deviations of the dependent variables were calculated. In the case of antibiotic use the 10^th^, 50^th^ and 90^th^ percentiles were used to statistically describe the different subgroups’ results (high prescribing and low prescribing PCCs respectively). As these statistical descriptions do not include the adjustments for explanatory factors the analysis was supplemented with the results of a linear mixed model including confidence intervals for repeated yearly observations in order to investigate for possible confounders. This mixed model can be split into two components: a “random” effect and a “fixed” effect. The random effect is that that PCCs have a random intercept (starting point) in their variables. In this way individual variation between the centres was considered. The fixed effect is manifested in all other parameters: type of ownership, geographical location, year etc. The residuals of this model based on annual data showed a homogeneity of variances and only little tendencies to skewness why the research group assessed that this model was viable in this study and refrained from using a multilevel model as all data was analysed at the level of the PCCs not aiming to attain any conclusions at the level of the individual citizen. The estimates, standard deviations and 95 % confidence intervals in this used model reveal the influence of the confounding factors, their variance and in which way results for the two groups change when adjusted for the confounding factors.

The mean patient perceived quality value was adjusted for CNI, number of listed citizens and location of each PCC as these factors showed influence in an earlier study [28]. The prescription rates of antibiotics and benzodiazepine derivatives were adjusted for CNI, proportion of gender groups and location of each PCC as these factors have shown influence earlier [18, 33, 34]. In the case of the antibiotic use the rate was also adjusted according to the proportion of age groups (0–19 years, 20–64 years and 65+) listed at PCC. For the outcome measures of the chronic diseases no adjustments were conducted as all PCCs were expected, according to regional guidelines, to carry out basic follow-up measurements as such as documentation of blood pressure and smoking habits regardless age, socioeconomic status, location or size of the PCC. In the few cases of missing data, PCCs were excluded from the calculations. All data was analysed using SPSS v22 and SAS v9.3.

### Ethical considerations

No data on individual patients was handled. According to Swedish law this aggregated data on the level of the PCC is public and not liable to any confidentiality which is why no ethical approval was necessary for this study.

## Results

### Descriptive data on the characteristics of the participating primary care centres

The characteristics of the PCCs studied are presented in Table 1: the 200 PCCs provided primary care services for 1.6 million citizens (99.7 % of the total population). 57 % (114/200) were publicly owned and 43 % (86/200) were privately owned. There were minor fluctuations with a small number of PCCs (*n* = 6) that closed down and or had just opened during the period studied but the total number of PCCs in the two compared groups remained stable with in sum only one additional privately owned PCC. From a geographical perspective 44 % of all PCCs were located within a 20 km range of the regional metropolis, an area where privately owned PCCs were overrepresented accounting for 54 % of all PCCs in that area. The population within this metropolitan area was younger compared with the population outside this area.

**Table 1:** Demographic characteristics of privately and publicly owned primary care centres (PCC)

	Privately owned PCC					Publicly owned PCC					all PCC				
	no. of PHCC	no. of listed citizens, (% of the pop.)		mean listed citizens/PHCC (SD)		no. of PHCC	no. of listed citizens, (% of the pop.)		mean listed citizens/PHCC (SD)		no. of PHCC	no. of listed citizens, (% of the pop.)		mean listed citizens/PHCC (SD)	
April 2011	86	510,123 (32.1 %)		5931.7 (3426.29)		114	1,075,225 (67.6 %)		9431.8 (3931.52)		200	1,585,348 (99.7 %)		7926.7 (4099.90)	
January 2014	87	580,304 (35.9 %)		6670.2 (3495.72)		114	1,032,359 (63.9 %)		9055.8 (3768,60)		201	1,612,663 (99.9 %)		8023.2 (3830.67)	
Change, (RG)	+1 (+1.1 %)	+70,181 (+12.1 %)		+738.5 (+11,1 %)		0 (0)	−42,866 (−4.2 %)		−375 (–4.2 %)		+1 (+0.5 %)	+27,315 (+1.7 %)		96.5 (+1.2 %)	
	geographic location: within or outside the regional metropolis, percentage of PCCs within their group														
		within		outside			within		outside			within		outside	
		56.9 %		43.1 %			34.5 %		65.5 %			44.0 %		56.0 %	
	gender in percentage of listed citizens														
		female		male			female		male			female		male	
April 2011		49.61 % (0.035)		50.39 % (0.035)			49.83 % (0.018)		50.17 % (0.018)			49.72 % (0.027)		50.28 % (0.027)	
January 2014		49.55 % (0.036)		50.45 % (0.036)			49.78 % (0.018)		50.22 % (0.018)			49.68 % (0.027)		50.32 % (0.027)	
	fraction of citizens within each group belonging to age groups (aged 0–19, 20–64, >64)														
	0–19		20–64		>64	0–19		20–64		>64	0–19		20–64		>64
April 2011	20.1 % (0.06)		62.4 % (0.08)		17.5 % (0.06)	23.4 % (0.04)		56.2 % (0.05)		20.4 % (0.05)	21.9 % (0.06)		58.9 % (0.08)		19.2 % (0.06)
January 2014	20.0 % (0.06)		62.4 % (0.08)		17.6 % (0.06)	23.4 % (0.04)		56.0 % (0.05)		20.6 % (0.05)	21.9 % (0.06)		58.8 % (0.07)		19.3 % (0.06)
	mean of Care Need Index														
April 2011	2.36 (0.912)					2.32 (0.632)					2.34 (0.762)				
January 2014	2.36 (1.034)					2.30 (0.667)					2.33 (0.844)				
	fraction of citizens within each group listed at PCC belonging to quintile 1–5 of Care Need Index (1 = most affluent, 5 = least affluent)														
	CNI Q1	CNI Q2	CNI Q3	CNI Q4	CNI Q5	CNI Q1	CNI Q2	CNI Q3	CNI Q4	CNI Q5	CNI Q1	CNI Q2	CNI Q3	CNI Q4	CNI Q5
	21 %	26 %	18 %	12 %	22 %	21 %	14 %	19 %	25 %	22 %	21 %	18 %	19 %	21 %	22 %
	Completeness of outcome measurement datasets for presciptions														
Prescription data of:	number of datasets		missing	completeness		number of datasets		missing	completeness		number of datasets		missing	completeness	
Antibiotics	2765		171	93.82 %		3732		117	96.86 %		6497		288	95.57 %	
Benzodiazepines (20–74yo)	2769		167	93.97 %		3732		117	96.86 %		6501		284	95.63 %	
Benzodiazepines (>74yo)	2743		193	92.96 %		3732		117	96.86 %		6475		310	95.21 %	
	Patient Perceived Quality (PPQ) (min 0- max 100 points)														
	PPQ dataset completeness 99.11 %					PPQ dataset completeness 96.25 %					PPQ dataset completeness 97.40 %				
	mean PPQ	recommend PCC		access	continuity	mean PPQ	recommend PCC		access	continuity	mean PPQ	recommend PCC		access	continuity
2011	82.4 (6.21)	86.3 (7.93)		84.7 (9.59)	70.4 (14.39)	79.6 (5.62)	81.7 (7.85)		80.18 (9.45)	58.2 (13.86)	80.8 (6.01)	83.6 (8.18)		82.1 (9.75)	63.2 (15.27)
2012	81.5 (6.35)	85.1 (9.03)		82.2 (10.47)	68.9 (14.14)	78.4 (5.63)	79.5 (8.34)		78.4 (9.47)	57.6 (14.01)	79.7 (6.12)	81.9 (9.04)		80.0 (10.05)	62.4 (15.11)
2013	81.4 (6.23)	84.7 (8.66)		78 (11.81)	68.1 (12.96)	77.9 (6.77)	79.0 (9.84)		77.5 (10.24)	57.1 (15.13)	79.4 (6.75)	81.4 (9.75)		77.9 (10.91)	61.8 (15.22)

Sample sizes, location and listed population with age, gender och socioeconomic characteristics. Completeness of datasets for drug prescription data. Results of the National Survey on Patient Perceived Quality (PPQ). Unless otherwise stated standard deviations in brackets. Abbreviations used in the table: *PCC* = primary care centre, no. = number, *pop* . = population, *SD* = standard deviation, *RG* = relative growth 2014 compared to 2011, *PPQ* = Patient Perceived Quality, *yo* = years old, *IHD* = Ischemic heart disease, *CNI Q1–5* Care Need Index quintile 1–5

The PCCs in the two groups differed considerably in the number of listed patients with a mean of approximately 5900 listed citizens at privately owned PCCs and 9400 listed citizens at publicly owned PCCs. During the period studied the mean size of the privately owned PCCs grew to 6700 listed citizens, while the mean size of the publicly owned PCCs shrank to 9100 listed citizens, reflecting the fact that the listed population at privately owned centres grew by 70,181 citizens, while the listed population at publicly owned centres decreased by 42,866 citizens (including a population growth in the county of 24,480 citizens).

There were only minimal differences between the two groups in the size of the gender groups of the listed citizens (difference 0.45 %). While the group of citizens of working age (aged 20–64) was steadily overrepresented at privately owned PCCs (62.4 % compared to 56.0 %), the groups of citizens aged 0–19 and over 64 showed a small but steady overrepresentation at publicly owned PCCs (23.4 % compared to 20 % for the younger group and 20.6 % compared to 17.6 % for the older group).

The socioeconomic indices of all PCCs varied mainly between CNI 1.03 (most affluent) and CNI 8.06 (least affluent) with a mean CNI of 2.33 in January 2014. The mean CNIs of the two groups of PCCs showed only small differences, but the group of privately owned PCCs had a higher variance in CNI (privately owned PCCs mean CNI 2.36, SD 0.912 vs. publicly owned PCCs mean CNI 2.32, SD 0.632) which indicates that their listed populations had a higher degree of segregation. As shown in Table 1 a comparison that also considered the different sizes of the PCC revealed for the group of privately owned PCCs that the fraction of citizens representing the second most affluent quintile was overrepresented at the cost of an underrepresentation of the fraction representing the second least affluent quintile (2^nd^ quintile 26 %, 4^th^ quintile 12 %). The reverse findings applied for the group of publicly owned PCCs (2^nd^ quintile 14 %, 4^th^ quintile 25 %).

**Fig. 1:**
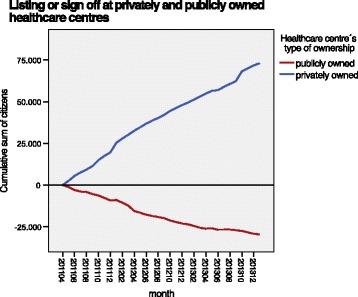
Listing or sign off at privately and publicly owned healthcare centres

**Fig. 2:**
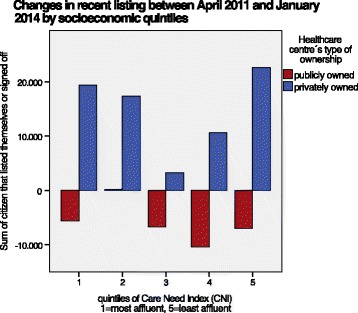
Changes in recent listing between April 2011 and January 2014 by socioeconomic quintiles

The observation of changes in the characteristics of the two groups during the period studied showed that the population growth in the county (27.315 citizens) and the transfer of citizens from publicly owned to privately owned PCCs (42.866 citizens) were continuous (Fig. 1). The majority of recently listed citizens at privately owned PCCs belonged to both the socioeconomically least affluent and most affluent populations, while the citizens who signed off from publicly owned centres mainly belonged to populations with average to low socioeconomic status (Fig. 2).

### Main results

#### Patient perceived quality

The unadjusted results of the national survey of patient perceived quality (Table 1) showed that the group of privately owned PCCs in 2011 received higher levels of satisfaction ratings than the group of publicly owned PCCs (mean value 82.4 compared to 79.6), especially in the item perceived continuity (70.4 compared to 58.2) and the item reflecting whether the patient would recommend the centre to others (86.3 compared to 81.7). Until 2013 the values decreased on average 1.2 % in both groups maintaining the order between the two groups. As confounding factors in the national survey of patient perceived quality are known through previous research, the results were adjusted for mean CNI, geographic location and size of the PCC. After adjustment, privately owned PCCs showed still higher values of mean patient perceived quality (Table 2). Populations that were less affluent and populations outside the regional metropolis tended towards a lower rating of patient perceived quality. The number of listed citizens of the PCC had also a small negative effect. However these results have to be interpreted with caution considering that the response rates of national survey of patient perceived quality are relatively low (51.3–53.4 %).

**Table 2:** Adjustments for Care Need Index, gender, age structure, size and location of primary care centre

Patient perceived Quality (mean)					Prescription rates of benzodiazepine derivates for patients aged 20–74				
Effect	Estimate	Standard error	95 % confidence interval		Effect	Estimate	Standard error	95 % confidence interval	
			Lower	Upper				Lower	Upper
Intercept	92.17	1.505	89.20	95.14	Intercept	−149.95	119.807	−385.27	85.36
Publicly owned	−2.24	0.858	−3.93	−0.56	Publicly owned	12.79	17.732	−22.03	47.62
Privately owned	0				Privately owned	0			
Year, 2011	1.10	0.568	−0.02	2.22	Year, 2011	−12.49	19.367	−50.53	25.55
Year, 2012	0.16	0.558	−0.94	1.26	Year, 2012	−5.42	18.869	−42.48	31.64
Year, 2013	0				Year, 2013	0			
Publicly owned * year, 2011	0.69	0.738	−0.76	2.14	Publicly owned * year, 2011	4.4	25.219	−45.14	53.93
Publicly owned * year, 2012	0.44	0.730	−0.99	1.88	Publicly owned * year, 2012	3.94	24.819	−44.8	52.69
Publicly owned * year, 2013	0				Publicly owned * year, 2013	0			
Privately owned * year, 2011	0				Privately owned * year, 2011	0			
Privately owned * year, 2012	0				Privately owned * year, 2012	0			
Privately owned * year, 2013	0				Privately owned * year, 2013	0			
CNI	−3.48	0.465	−4.40	−2.57	CNI	22.01	6.986	8.29	35.73
within regional metropolis	−1.78	0.732	−3.23	−0.34	Proportion female	821.64	234.167	361.7	1281.58
outside regional metropolis	0				Proportion male	0			
number of listed citizens (in 100)	−0.03	0.010	−0.05	−0.01	within regional metropolis	−50.99	10.714	−72.03	−29.95
					outside regional metropolis	0			
					Prescription rates of benzodiazepine derivates for patients aged over 74				
Prescription rates of antibiotics									
Effect	Estimate	Standard error	95 % confidence interval						
			Lower	Upper					
Intercept	5.89	2.426	1.13	10.66					
Publicly owned	−1.3	0.271	−1.83	−0.77	Effect	Estimate	Standard Error	95 % confidence interval	
Privately owned	0							Lower	Upper
Year, 2011	0.89	0.287	0.33	1.46	Intercept	602.43	450.151	−281.74	1486.59
Year, 2012	0.89	0.280	0.34	1.44	Publicly owned	50.98	66.624	−79.88	181.84
Year, 2013	0				Privately owned	0			
Publicly owned * year, 2011	−0.49	0.374	−1.23	0.24	Year, 2011	−201.67	72.766	−344.59	−58.75
Publicly owned * year, 2012	−0.43	0.368	−1.15	0.3	Year, 2012	−135.76	70.896	−275	3.49
Publicly owned * year, 2013	0				Year, 2013	0			
Privately owned * year, 2011	0				Publicly owned * year, 2011	134.16	94.756	−51.96	320.27
Privately owned * year, 2012	0				Publicly owned * year, 2012	76.66	93.253	−106.5	259.82
Privately owned * year, 2013	0				Publicly owned * year, 2013	0			
CNI	0.57	0.109	0.36	0.78	Privately owned * year, 2011	0			
Proportion female	3.8	3.555	−3.18	10.78	Privately owned * year, 2012	0			
Proportion male	0				Privately owned * year, 2013	0			
Proportion of 0-19	0.95	1.877	−2.73	4.64	CNI	119.38	26.248	67.83	170.94
Proportion of 20-64	−5.19	1.811	−8.74	−1.63	Proportion female	1637.02	879.835	−91.1	3365.14
Proportion of 65+	0				Proportion male	0			
within regional metropolis	−0.8	0.183	−1.15	−0.44	within regional metropolis	212.03	40.256	132.96	291.1
outside regional metropolis	0				outside regional metropolis	0			

The estimates of the mean patient perceived quality, prescription rates of antibiotics and benzodiazepine derivatives per ownership type are calculated by a linear mixed model for repeated yearly observations (for the 197 PCCs with data during 2011–2013). Adjustments were made selectively for year, location, number of listed citizens (in 100), Care Need Index, proportion of gender group listed and proportion of age groups listed at PCC (0–19 years, 20–64 years and 65+). * indicates the interaction of two effects

#### Rate of purchased antibiotics

**Fig. 3:**
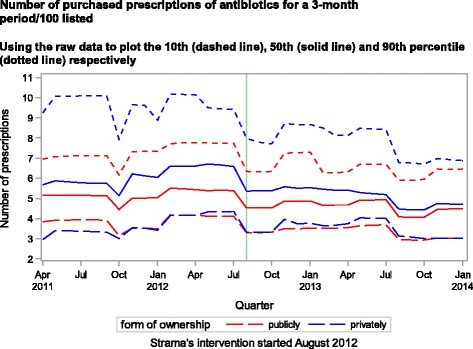
Number of purchased prescriptions of antibiotics for a 3-month period/100 listed. Using the raw data to plot the 10th (dashed line), 50th (solid line) and 90th percentile (dotted line) respectively

The mean rate of purchased antibiotics prescribed by the group of privately owned PCCs (6.0 purchased prescriptions per quarter/100 listed citizens, SD 2.78) was steadily higher and had a larger variance than the mean rate of the group of publicly owned PCCs (5.1 purchased prescriptions per quarter/100 listed citizens, SD 1.50). Differences between the groups were largest in 2011 and decreased over time (Fig. 3). While the 10^th^ percentile of each group had rates that were similar, differences increased in the comparison of the 50^th^ to the 90^th^ percentile. Rates decreased for both groups and all subgroups but especially for the 90^th^ percentile of the group of privately owned PCCs after August 2012. Table 2 illustrates the finding that the group of publicly owned PCCs had lower rates compared to the group of privately owned PCCs, even when adjusted for mean CNI, gender, age structure and geographic location of the PCC.

#### Rates of purchased benzodiazepine derivatives

The rates of purchased benzodiazepine derivatives for elderly aged over 74 were in general more than fivefold higher than those for younger individuals aged 20–74 (on average 1771.17 vs. 293.81 DDD/100 patients/month). These internationally compared already high rates increased for all PCCs from 2011 to 2013 by 3.6 % for younger individuals and by 7.4 % for elderly individuals (Table 3). The group of privately owned centres showed a steadily lower mean of the rates for younger individuals with a higher variation than the group of publicly owned centres (277.98 DDD/100 patients/month, SD 151.66 vs. 305.56 DDD/100 patients/month, SD 103.46). The use of benzodiazepine derivatives for the elderly showed the same tendency between the two groups in 2011 but the relative differences diminished over time due to the fact that the group of privately owned PCCs increased their prescription rates from 2011 to 2013 more than twice as much as the group of publicly owned centres (increase rates 11.5 % vs. 4.7 %). Variations in rates of purchased benzodiazepine between PCCs were in general large. Table 2 illustrates the fact that the group of privately owned PCCs showed a lower use of benzodiazepine derivatives even when adjusted for mean CNI, gender and geographic location of the PCC. However, the 95 % confidence interval reveals substantial variance. Rates for younger patients tended to be higher outside the regional metropolis, while the rates for older patients showed the reverse results.

**Table 3:** Prescription of benzodiazepine derivates

		For individuals aged between 20–74				For individuals >74 years of age			
Year	Type of owner-ship	Mean DDD	Standard deviation	Increase- rate compared to 2011	Deviation from mean DDD for all PCCs	Mean DDD	Standard deviation	Increase-rate compared to 2011	Deviation from mean DDD for all PCCs
2011	publicly	299.20	103.04		4.83 %	1751.85	445.74		2.70 %
	privately	266.64	162.59		−6.58 %	1643.16	740.16		−3.68 %
	all PCCs	285.42	132.49			1705.88	590.77		
2012	publicly	309.19	105.49	3.34 %	3.91 %	1775.69	440.47	1.36 %	1.23 %
	privately	281.73	151.34	5.66 %	−5.32 %	1724.35	578.87	4.94 %	−1.70 %
	all PCCs	297.55	127.65	4.25 %		1754.10	503.86	2.83 %	
2013	publicly	306.22	101.55	2.35 %	3.55 %	1833.08	433.27	4.64 %	0.03 %
	privately	281.83	144.04	5.70 %	−4.70 %	1831.66	567.52	11.47 %	−0.04 %
	all PCCs	295.74	122.21	3.61 %		1832.47	494.94	7.42 %	
Total	publicly	305.56	103.46		4.00 %	1790.97	440.33		1.12 %
	privately	277.98	151.66		−5.39 %	1744.23	623.95		−1.52 %
	all PCCs	293.81	126.98			1771.17	526.45		

Purchased prescriptions in defined daily doses (DDD) per 100 listed individuals at PCC, divided into age-group, year and type of ownership

#### Follow-ups carried out for patients with chronic diseases

The group of privately owned PCCs showed in comparison with the group of publicly owned PCCs lower rates of follow-ups carried out for patients with HPT (66.6 % vs. 70.9 %), IHD (63.6 % vs. 67.9 %) and DM (83.4 % vs. 85.0 %) and higher variation for all three chronic diseases (Table 4). All rates of follow-ups carried out for patients with DM showed improvements over time regardless of ownership type; particularly the follow up for urine micro-albumin improved in the group of privately owned PCCs from 59.4 to 70.6 %. Rates of blood pressure measurements carried out for all three chronic diseases were between 82.5 and 94.5 %. Rates of the documentation of smoking habits for patients with IHD and HPT were only at 50.8–59.1 % yet the same rates for patients with DM were 84.9–89.7 %. Rates of the documentation of LDL measurements were between 67.9 and 77.0 % for patients with DM, and 55.3–60.5 % for patients with IHD.

**Table 4:** Percentage of documented follow-ups carried out for certain chronic diseases

Chronic disease	Patients with documentation for	Year	Privately owned PCC			Publicly owned PCC		
			Percentage mean	SD	Percentage data completeness	Percentage mean	SD	Percentage data completeness
Diabetes mellitus (DM)	HbA1c measurement	2011	91.1	8.53	98.8	90.8	8.06	100.0
		2012	92.9	7.63	100.0	92.5	5.20	100.0
		2013	94.5	5.40	98.8	94.2	5.78	100.0
	blood pressure	2011	90.7	7.99	98.8	90.2	7.80	100.0
		2012	92.9	8.09	100.0	92.9	4.26	100.0
		2013	93.6	7.19	98.8	94.5	4.17	100.0
	body mass index	2011	82.9	12.96	98.8	84.4	9.96	100.0
		2012	92.9	12.01	100.0	87.3	7.68	100.0
		2013	93.6	9.07	98.8	89.8	6.79	100.0
	smoking status	2011	84.9	12.64	98.8	85.4	10.73	100.0
		2012	89.1	11.39	100.0	87.9	8.79	100.0
		2013	88.9	9.46	98.8	89.7	8.97	100.0
	micro-albumin urine test	2011	59.4	24.54	98.8	74.2	17.73	100.0
		2012	68.4	22.49	100.0	75.6	17.28	100.0
		2013	70.6	19.3	98.8	76.7	16.46	100.0
	low-density lipoprotein	2011	67.9	16.75	98.8	71.4	14.49	100.0
		2012	71.6	17.36	100.0	74.9	13.67	100.0
		2013	76.1	13.91	98.8	77.0	12.89	100.0
	average percentage		83.4	12.60	99.2	85.0	10.04	100.0
Ischemic heart disease (IHD)	blood pressure	2012	82.5	8.27	89.7	84.1	6.23	99.1
	smoking status	2012	53.0	17.52	78.9	59.1	14.06	98.2
	low-density lipoprotein	2012	55.3	16.20	71.6	60.5	12.94	99.1
	average percentage		63.6	14.00	80.1	67.9	11.08	98.8
Hypertension (absence of DM and IHD)	blood pressure	2012	82.4	9.14	96.4	83.9	7.45	99.1
	smoking status	2012	50.8	21.12	93.8	57.8	14.5	99.1
	average percentage		66.6	15.13	95.1	70.9	10.98	99.1

*SD* = standard deviation

## Discussion

This study shows varying differences between the groups of privately and publicly owned PCCs in patient perceived quality, antibiotic use, the use of benzodiazepine derivatives and the rates of follow-ups carried out for patients with chronic diseases. However the findings of this study cannot unambiguously answer the question of whether or not the quality of primary healthcare services is influenced by the healthcare centre’s type of ownership: while the group of privately owned PCCs rate higher in patient perceived quality and have a lower use of benzodiazepine derivatives, the group of publicly owned PCCs show a lower use of antibiotics and have higher rates of follow-ups carried out for patients with chronic diseases. At the same time this study revealed the existing structural differences between these two groups of primary care providers: Privately owned PCCs were on average smaller, to a higher degree situated in the regional metropolis, had higher fractions of citizens within the second most affluent socioeconomic quintile and an overrepresentation of citizens of working age. In their turn, publicly owned PCCs were on average larger, to a higher degree situated outside the regional metropolis, had higher fractions of citizens within the second least affluent socioeconomic quintile and had an overrepresentation of juveniles and elderly.

The reader needs to consider an important limitation of this study as it is based on aggregated data and not on individual data, and as the differences between the two types of ownership were only analysed on the aggregated level for the whole county and do not include a multilevel analysis. Even if the location of the PCC (metropolitan vs. rural) was taken into account in the adjustments for confounding factors the geographical differences between the various parts of the county were not investigated. These limitations mean that there is a risk of an ecological inference fallacy and that conclusions can neither be drawn for any individual nor for any subarea of the county according the quality of healthcare services as there were variations within the two groups studied, as well as in each organizational unit. These variations might lead to completely different interpretations at the level of an individual or the level of a subarea compared to the aggregated level of the county. The application of a linear-mixed model based on annual data was considered viable in this study as the residuals of this model showed a homogeneity of variances. However, due to the fact that a model on the basis monthly data would have shown relevant skewness, further methodological improvements should be considered: If logarithmized, data becomes less skewed and the geometric mean (the mean of the logarithmized data of all observations) becomes an estimate of the median. However, this does not provide information about certain subgroups of interest, i.e. PCCs with high prescription rates. Therefore further research is necessary including the development of a viable model using quantile regression. The research team addressed this problem in this study by choosing raw data plots to illustrate the prescription rates of antibiotics, being aware that this figure does not include the adjustment for explanatory factors and needed to be supplemented with the results of the above-mentioned mixed model.

Certain methodical weaknesses occur due to the imprecision of variables such as the fact that this study does not count the actual number of prescriptions made at a PCC but uses a proxy measures to describe the use of antibiotics and benzodiazepine derivatives at a PCC: through data from the Swedish eHealth Agency the number of purchased drugs which were prescribed at a certain PCCs is calculated, meaning that actual prescriptions rates can be higher as not all patients purchased the prescribed drugs. Datasets for antibiotic use were neither adjusted for age nor gender and nor was it necessarily connected to the patients listed at the PCC. This was partially addressed by adjustments for gender and age as higher rates of antibiotic use have been reported for the elderly and for females. It was not possible to adjust for some influencing factors such as the PCCs location, as PCCs on the coast had considerably higher prescription rates in summer caused by the number of tourists [34]. Further imprecision might have occurred as some GPs have used their PCC workplace code incorrectly during Out-of-hours services leading to spuriously higher rates of antibiotic use for their ordinary PCCs. With the regular publications via the Swedish Strategic Programme Against Antibiotic Resistance (Strama) in Region Västra Götaland, awareness of this error has increased among prescribers during the period studied, and thus the decrease in prescription rates at a few individual PCCs could be partly caused by a more correct use of the workplace code. Studies that were not susceptible to this as they included antibiotic prescriptions from all different prescribers support the finding of a generally decreased antibiotic use in the county [34]. The rates of purchased prescriptions of benzodiazepine derivatives could be biased by the fact that a pattern of many short patient visits at a PCC would reduce the rate due to the fact that it was the number of patients visiting the PCC that was counted in the denominator and not the number of listed citizens. While the datasets on DM and HPT generally showed high completeness, the datasets on IHD in the group of privately owned PCCs had a lower completeness level that is obstructive to the comparison. Technical obstacles leading to some missing data during the collection and aggregation of data at the regional healthcare authorities weaken the results of the study to some extent. Through the exclusion of Out-of-office services and single-handed practices due to lack of available data it was impossible to include all purchased prescriptions for the whole of primary care. Although methodical limitations of the national patient survey have been discussed in earlier reports, including the low patient response rates between 51.3 and 53.4 % and the influence of socioeconomic and geographic factors, the results were valuable to some extent in the context of this group comparison [28].

On the other hand certain strengths in this study allowed researchers to obtain an extensive picture of quality of primary care services in the county studied. These strengths were the high completeness rates of almost all datasets and the inclusion of nearly the whole population. Furthermore as many as possible modifying factors for outcomes of the populations have been included in the adjustments such as the socioeconomic index, variations in gender and age structure and the size and location of the PCCs.

The finding that the group of privately owned PCCs steadily showed higher patient perceived quality than the group of publicly owned PCCs (even after adjustment for confounders) seems consistent with the finding that there was concurrently a continuous shift of patients from publicly owned PCCs to privately owned PCCs indicating that citizens who were less satisfied with the primary care services at publicly owned PCCs chose to register at privately owned PCCs (even if the results of the national patient survey have to be interpreted with caution due to methodical limitations). These findings of this study differ from a prior national study that included three counties (one third of all PCCs in Sweden) and revealed that privately owned PCCs received higher ratings in patient perceived quality than publicly owned PCCs but that this difference vanished after adjustment for the socioeconomic factors and the morbidity burden [28]. In general these findings have to be interpreted with caution as previous research in an international context showed a number of methodological problems and unresolved issues in patients perceived quality of care such as ceiling effects; uncertainty about whether instruments are reliable and valid across cultures and the continuing reliance in many surveys on ratings in which expectations are confounded with experiences [35].

In order to achieve a more rational use of antibiotics with a goal of 250 purchased prescriptions per 1000 inhabitants and year, Strama pronounced in the Region Västra Götaland a theoretical goal of an average for all PCCs of 3.1 purchased prescriptions/100 listed citizens and quarter. Due to varying preconditions, individual PCCs may have differing rates although guidelines for a rational antibiotic use are followed. However, the observed variation with the maximum rates at individual privately owned PCCs of up to ten times and at individual publicly owned PCCs of up to four times of the average theoretically aimed at, indicates that PCCs were either over-prescribing antibiotics or providing care for an exceptionally high number of patients with infectious diseases. The latter might be explained by an uneven distribution of these patients, which though is in conflict with the PCC’s task to primarily provide care for their listed population. The findings of this study show that the use of antibiotics and the variations between PCCs decreased after August 2012 particularly for high-prescribing PCCs. Concurrently with the decrease, Strama became part of the regional administration in Västra Götaland and started an intervention lasting throughout the rest of the period studied. Beside information about antibiotic resistance, antibiotic prescribing guidelines and current prescribing patterns, it included the involvement of one General Practitioner (GP) from each PCC as a key person who initiated meetings with colleagues for the purpose of reflection [34]. Two behavioural studies conducted recently that investigated factors influencing Swedish GPs’ use of antibiotics found four factors that supported a rational use: forums at PCCs for discussion on guidelines; leadership and support to local opinion leaders; inter-professional collaboration; and opportunities for professional development [36]. These findings support the assumption that Strama’s intervention had an important impact on the greatest decrease nationwide of antibiotic prescriptions which occurred in the county during the period studied and which is well in accordance to the WHO’s call for action against antimicrobial resistance. Compared internationally Sweden has a rather low and moreover decreasing rate of antibiotic use and has one of the lowest antibiotic resistance rates [37]. A recent study showed that poor governance and corruption–two factors under good control in Sweden - contribute beside usage volumes to the level of antibiotic resistance [38]. The same study also showed, without having a clear explanation, positive correlation between the percentage of private health expenditure in a country and the degree of antibiotic resistance, presuming in this sector fewer controls on broad-spectrum agents, the length of time of drug therapy and the volumes used.

The alarming finding that the already high rates of purchased prescriptions of benzodiazepine derivatives further increased in both groups, especially for elderly patients who are prone to several adverse effects, stands in contrast to the reduced use of antibiotics. But it corresponds well to earlier investigations that showed that GPs are averse to addressing the public health problem of benzodiazepine overuse in the elderly. GPs endorsed benzodiazepines as effective treatment for anxiety, citing quick action and strong patient satisfaction [39]. They stated as causes limited physician time and poor reimbursement for Mental Health Care as causes and complained about a lack of training in constructive strategies to address anxiety problems [39, 40]. A recent review showed that supervised benzodiazepine withdrawal augmented by psychotherapy should be considered in older people–a resource-intensive therapy that stands in conflict with the situation of competing PCCs aiming to provide care for as many patients as possible [41]. Educational outreach visits on prescribing benzodiazepines to elderly in southern Sweden were effective in modifying GPs’ prescribing habits [42]. The absence of a comparable comprehensive education program might explain the contradictory trends in the use of antibiotics and benzodiazepines. In order to address this unfavourable trend further investigations and evidence-informed interventions are necessary.

The findings concerning the rates of follow-ups carried out for patients with DM, IHD and HPT do not support the assumption that physicians might tend to register diagnoses in order to benefit from economic incentives although no adequate follow-up of the condition was carried out, which has been discussed in other settings [43]. Smaller variations between PCCs should be acceptable as reasonable practice forbids a strict adherence to guidelines particularly for the elderly as other questions related to person-focused care (e.g. quality at the end of life) can become more important and the allocation of resources must be considered wisely [44]. However this study does not contradict earlier findings that change of reimbursement system elevates rates of diagnoses [45].

The two different quality registries involved (NDR and QregPV) have been in existence for different lengths of time. NDR, which is nationwide and has been established for longer shows generally higher rates of follow-ups carried out. This indicates a need for further research on how the acceptance and usage of a registry influences healthcare routines [46].

In recent years patients’ and medical professionals’ perceptions, patients’ opportunities for informed choices and the effects of patients’ choices associated with the reforms in Swedish primary healthcare have been studied [2, 6, 9, 28]. The findings of this study revealed, even if it cannot unambiguously answer the question of whether or not the quality of primary healthcare services is influenced by the healthcare centre’s type of ownership, that the two groups differ structurally (geography and size of the PCCs) and in the composition of the population served. Additionally the variation in outcome measures was higher at privately owned PCCs. Furthermore, both favourable and disadvantageous trends occur simultaneously and the patient perceived quality was on a group level not consistent with the other outcome measures. As healthcare in European countries in the recent years has become to some extent more private, studies have been carried out to evaluate the effects [47]. However, a recent review stated that the evidence for recurring privatisation questions is weak and mixed [48]. The public-private mix in Swedish primary healthcare has changed substantially with consequences difficult to predict while the lack of data and neglect of research in this field have hindered informed policy-making [49]. It can be questioned if the competition- and incentive-oriented approach of the recent Swedish reforms can contribute to sustainable improvements in the quality of primary healthcare services. The unequal distribution of the population (concerning socio-economy and geography and age) to the two different groups of primary care providers correspond well to prior findings like the increased probability of choosing a publicly owned PCC instead of a privately owned one, the older the individual and the higher the comorbidity level [50]. These effects were certainly not intended by the healthcare reform and their impact on future trends including other effects on i.e. recruitment of medical professionals remains unclear. Future reforms that aim to create an effective and sustainable primary care system should therefore be evidence-informed and continuously evaluated through close cooperation of health service researchers and policy makers.

## Conclusions

The findings of this study cannot unambiguously answer the question of whether or not the quality of primary healthcare services is influenced by the healthcare centre’s type of ownership. However they question the political prediction that the most recent healthcare reform including a shift of power from officials to citizens and an increase of providers and their diversity would create conditions that encourage care providers to improve the quality and efficiency of care. As the quality of primary healthcare services showed concurrently improvements and impairments in the different studied outcomes it can be questioned whether the competitive environment encouraged quality improvements. The detected tendencies of an unintended unequal distribution of the population between the two types of ownership with disparities in age-groups, socio-economy and geography might imply unpredictable consequences for the recruitment of scarce health care professionals (e.g. General Practitioners) and thus risk an equal care provision. A continuous observation of effects and further studies and are needed to elucidate possible causal relations and to enable evidence-informed policy-making.
